# Supplementing electronic health records through sample collection and patient diaries: A study set within a primary care research database

**DOI:** 10.1002/pds.4323

**Published:** 2017-09-19

**Authors:** Rebecca M. Joseph, Jamie Soames, Mark Wright, Kirin Sultana, Tjeerd P. van Staa, William G. Dixon

**Affiliations:** ^1^ NIHR Manchester Musculoskeletal Biomedical Research Unit Central Manchester University Hospitals NHS Foundation Trust, Manchester Academic Health Science Centre Manchester UK; ^2^ Arthritis Research UK Centre for Epidemiology, Centre for Musculoskeletal Research, Institute of Inflammation and Repair, Faculty of Medical and Human Sciences, Manchester Academic Health Science Centre University of Manchester Manchester UK; ^3^ Clinical Practice Research Datalink Medicines and Healthcare Products Regulatory Agency London UK; ^4^ Health eResearch Centre, Farr Institute for Health Informatics Research University of Manchester UK; ^5^ Rheumatology Department Salford Royal NHS Foundation Trust Salford UK; ^6^ NIHR Manchester Biomedical Research Centre Central Manchester NHS Foundation Trust, Manchester Academic Health Science Centre UK

**Keywords:** electronic health records, nested design, observational study, pharmacoepidemiology, sample collection

## Abstract

**Purpose:**

To describe a novel observational study that supplemented primary care electronic health record (EHR) data with sample collection and patient diaries.

**Methods:**

The study was set in primary care in England. A list of 3974 potentially eligible patients was compiled using data from the Clinical Practice Research Datalink. Interested general practices opted into the study then confirmed patient suitability and sent out postal invitations. Participants completed a drug‐use diary and provided saliva samples to the research team to combine with EHR data.

**Results:**

Of 252 practices contacted to participate, 66 (26%) mailed invitations to patients. Of the 3974 potentially eligible patients, 859 (22%) were at participating practices, and 526 (13%) were sent invitations. Of those invited, 117 (22%) consented to participate of whom 86 (74%) completed the study.

**Conclusions:**

We have confirmed the feasibility of supplementing EHR with data collected directly from patients. Although the present study successfully collected essential data from patients, it also underlined the requirement for improved engagement with both patients and general practitioners to support similar studies.

## 1 INTRODUCTION

United Kingdom (UK) primary care electronic health records (EHR) are a valuable data source for epidemiological research as they contain a broad range of prospectively collected data for large samples of the population. However, because the purpose of data collection is routine health care delivery, certain information relevant to specific research questions may not be captured. Such questions would therefore require an alternate data source, or for primary care EHR to be supplemented with the missing information.

In this brief report, we describe a study in which new data were collected directly from patients to supplement EHR data. This builds on prior examples such as the STAGE study^1^, ^2^ that demonstrated the feasibility of supplementing primary care EHR from the Clinical Practice Research Datalink (CPRD)^3^ with genetic data.

The purpose of our study was to investigate adrenal insufficiency following glucocorticoid exposure in patients with rheumatoid arthritis (RA). Adrenal insufficiency, which has non‐specific symptoms,^4^, ^5^ is likely to be under‐reported or misclassified in the EHR. Additionally, prescription data may differ from true drug exposure due to factors such as nonadherence.^6^ We therefore collected saliva samples from participants, using cortisol levels to define adrenal insufficiency, and collected information about glucocorticoid exposure using a patient‐reported diary. We describe the study methodology, present the recruitment rate and success of sample collection, and discuss the limitations.

**KEY POINTS**

- We have provided further evidence supporting the feasibility of supplementing electronic health records (EHR) with patient derived data.
- Supplementing the EHR may address possible misclassification and/or missing information within EHR
- Challenges in practice and patient recruitment demonstrated the importance of considering ways to maximise recruitment.

## 2 METHODS

This was an observational study set within English primary care. Participants were recruited between September 2015 and April 2016. Ethical approval was granted by the National Research Ethics Service Committee (reference 14/LO/1335) and the Independent Scientific Advisory Committee for use of CPRD data (reference 14_145R).

### 2.1 Study population

The search criteria based on the following inclusion and exclusion criteria were applied to the full CPRD dataset. Inclusion criteria were: (1) diagnosis of RA (defined using a validated algorithm^7^), (2) age 16 or over, (3) registered at an English general practice, and (4) prescribed oral glucocorticoids within the last 2 years. Exclusion criteria were adrenal insufficiency unrelated to glucocorticoid use, other condition or treatment with the potential to affect adrenal function, or less than 2 years of data within CPRD. The list was generated in June 2015 and updated in December 2015. General practitioners were asked to screen the list of patients to confirm eligibility and exclude patients they judged unsuitable (eg, unable to give consent based on English‐language information sheets, recent bereavement). All participants gave their consent to take part in the study. We aimed to recruit 400 participants.

Based on the search performed in December 2015, there were 19 665 patients with RA who were currently active in practices contributing to CPRD and registered at an English general practice. Of these patients, 50% had never used oral GCs, 29% had not used oral GCs within the last 2 years, and approximately 1% were excluded for having less than 2 years of data within CPRD or having a condition known to affect the adrenal glands. The remaining 3974 (20%) were the population considered potentially eligible for inclusion in the study.

### 2.2 Practice recruitment

General practices were responsible for mailing invitations to patients as only general practices are able to identify their patients from the EHR. To recruit practices, an initial invitation letter and expression of interest form was sent to each of the 252 practices in England with eligible patients. If practices did not respond, they were followed up with another postal invitation, an email, and a final postal reminder. Costs to practices were minimised: patient invitation materials were provided pre‐prepared to practices, and the practices were reimbursed for their time by the National Institute for Health Research (NIHR) Clinical Research Network (CRN).

### 2.3 Study protocol

Invitation packs containing a letter from the general practice, an information sheet, and a consent form were mailed to eligible patients by their general practices. Patients who wished to take part were asked to complete and return the consent form, along with their contact details, to the research team at the University of Manchester.

Study materials were mailed to all patients who returned a valid consent form. On a morning of their choice, participants were instructed to provide saliva samples and complete a diary about recent glucocorticoid use. The samples and diary were mailed to the researchers. After analysis, patients with a low salivary cortisol level were followed up by letter. With permission, letters were also sent to their GP.

After all patients were followed up, the study data were anonymised. CPRD then provided the EHR data for the study participants, with CPRD identifiers replaced with the participants' study IDs. At no point was it possible for the research team or CPRD to link identifiable information to the EHR.

## 3 RESULTS

### 3.1 Practice recruitment

All 252 practices with eligible patients in August 2015 were invited to participate. Of these, 101 (40%) practices responded after the first invitation, 47 (19%) after at least 1 reminder, and 104 (41%) never responded. In total, 77 (31%) practices expressed interest in being involved and 71 (28%) declined the invitation. Sixty‐six practices (26% of 252) completed the mail‐out to patients.

### 3.2 Patient recruitment

**Figure 1:**
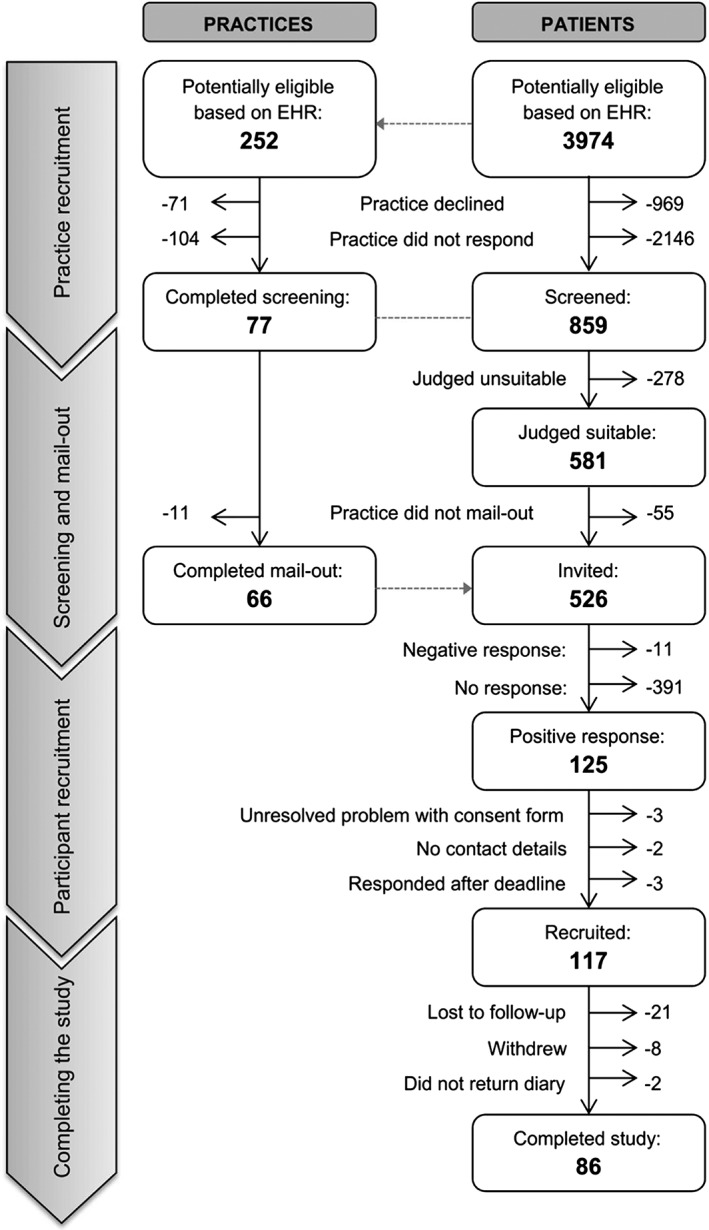
**Flow of potentially eligible patients and their practices through the study.** Corresponding practice numbers are shown for clarity. The phase of the study is indicated by the column on the left. Abbreviations: EHR, electronic health records

Of the 3974 patients considered potentially eligible for inclusion in the study, 859 (22%) were registered with one of the 77 practices that agreed to take part. Invitations were sent to 526 patients, and 117 patients returned valid consent forms. The median time from practices mailing invitations to participants being recruited was 25 (range 6–149) days. All recruited participants were sent diaries and sample collection kits: we had no further contact from 21 participants and 8 participants withdrew (all before returning saliva samples). The flow of patients through the study is presented in Figure 1.

### 3.3 Data collection

In total, 86 participants returned both saliva samples and diaries, and 2 participants returned saliva samples but not diaries. The median time from mailing study materials to receiving the saliva samples was 12 (range 5–127) days. Four of the samples could not be analysed: 3 of the collection tubes were empty, and 1 sample was omitted from the batch (in error).

## 4 DISCUSSION

In this study, we were able to collect saliva samples and self‐reported drug‐use information from 86 participants to supplement EHR data. The new data collected will allow us to define the study outcome, adrenal insufficiency, more accurately than using primary care EHR data alone, as symptoms of adrenal insufficiency are non‐specific and many cases are only diagnosed if patients present as emergencies.^4^, ^5^ The self‐reported drug use data will allow us to quantify misclassification in exposure to oral glucocorticoids and adjust the analyses accordingly. However, the final study population was small, and we did not reach our recruitment target of 400.

Practice recruitment was a major limiting factor for our final participant figures—only 26% of general practices with eligible patients sent invitations to patients. The STAGE study also report practice recruitment as a limit on patient recruitment, although at 53%, the rate of practice recruitment was higher than in our study.^1^ PLEASANT, a later study conducted by CPRD which only required practices to mail a letter to patients, did recruit their target of 140 practices over a 7‐month period.^8^ This total included 129 of the 433 practices invited by CPRD (30%). Reaching the target number of practices required significant staff resource to follow up the practices.^8^ General practices are currently experiencing high and increasing time and financial pressures.^9^ Aside from frequently following up practices, researchers could make use of primary care study tools and platforms such as FARSITE (NorthWest Ehealth) and TrialBase (CPRD), which help streamline the research process, to encourage practice participation.

The proportion of patients who were recruited was also small—117 (22%) were recruited and 86 (16%) completed the study out of 526 invited. This recruitment rate was lower than that of the STAGE study, which used a similar methodology yet had a recruitment rate of 34% (754 of 2194).^1^ Recruitment for STAGE was over a much longer period (36 months compared with 8 months). In addition, recruitment rates were higher for patients asked to provide a blood sample, at their local general practice, than patients asked to provide saliva samples in their homes.^1^ Recruitment of participants is a challenge common to all research studies. Suggestions for increasing participation in research discussed in the literature include providing incentives, improving communication with patients about the study, and minimising the burden for participants.^10^ Greater patient and public involvement from the outset of a study may also help improve recruitment.^11^

In conclusion, we have demonstrated that sample collection and patient diaries can be nested with primary care EHR research databases. Almost all (84 of 87 tested) of the saliva samples collected for our primary outcome were analysed successfully and provide data which is not available in the EHR.

## ETHICS STATEMENT

Ethical approval was granted by the National Research Ethics Service Committee (reference 14/LO/1335) and the Independent Scientific Advisory Committee for use of CPRD data (reference 14_145R).

## CONFLICT OF INTEREST

The study sponsor had no involvement in study design, in the collection, analysis and interpretation of data, in writing the manuscript, or in the decision to submit the manuscript for publication.

TPvS has received grants and personal fees from GSK and personal fees from Sanofi, Roche, and NovoNordisk outside the submitted work.

JS, MW, and KS are employees of CPRD.

There are no further conflicts to declare.

## References

[1] O'Meara H , Carr DF , Evely J , et al. Electronic health records for biological sample collection: feasibility study of statin‐induced myopathy using the Clinical Practice Research Datalink. Br J Clin Pharmacol. 2014;77(5):831‐838. https://doi.org/10.1111/bcp.12269 24308359 10.1111/bcp.12269 PMC4004403

[2] Carr DF , O'Meara H , Jorgensen AL , et al. SLCO1B1 genetic variant associated with statin‐induced myopathy: a proof‐of‐concept study using the clinical practice research datalink. Clin Pharmacol Ther. 2013;94(6):695‐701. https://doi.org/10.1038/clpt.2013.161 23942138 10.1038/clpt.2013.161 PMC3831180

[3] Clinical Practice Research Datalink . http://www.cprd.com. 2017. Accessed 03 Apr, 2017.

[4] Burton C , Cottrell E , Edwards J . Addison's disease: identification and management in primary care. Br J Gen Pract. 2015;65(638):488‐490. https://doi.org/10.3399/bjgp15X686713 26324491 10.3399/bjgp15X686713 PMC4540394

[5] Arlt W , Allolio B . Adrenal insufficiency. The Lancet. 2003;361(9372):1881‐1893. https://doi.org/10.1016/s0140‐6736(03)13492‐7 10.1016/S0140-6736(03)13492-7 12788587

[6] Tamblyn R , Eguale T , Huang A , Winslade N , Doran P . The incidence and determinants of primary nonadherence with prescribed medication in primary care: a cohort study. Ann Intern Med. Apr 1 2014;160(7):441‐450. https://doi.org/10.7326/M13‐1705 24687067 10.7326/M13-1705

[7] Thomas SL , Edwards CJ , Smeeth L , Cooper C , Hall AJ . How accurate are diagnoses for rheumatoid arthritis and juvenile idiopathic arthritis in the general practice research database? Arthritis Rheum. 2008;59(9):1314‐1321. https://doi.org/10.1002/art.24015 18759262 10.1002/art.24015

[8] Horspool MJ , Julious SA , Mooney C , May R , Sully B , Smithson WH . Preventing and lessening exacerbations of asthma in school‐aged children associated with a new term (PLEASANT): recruiting primary care research sites‐the PLEASANT experience. NPJ Prim Care Respir Med. 2015;25:15066 https://doi.org/10.1038/npjpcrm.2015.66 26562491 10.1038/npjpcrm.2015.66 PMC4642399

[9] Baird B , Charles A , Honeyman M , Maguire D , Das P . Understanding Pressures in General Practice. The King's Fund: London, UK; 2016.

[10] Bower P , Brueton V , Gamble C , et al. Interventions to improve recruitment and retention in clinical trials: a survey and workshop to assess current practice and future priorities. Trials. 2014;15(1):399 https://doi.org/10.1186/1745‐6215‐15‐399 25322807 10.1186/1745-6215-15-399 PMC4210542

[11] Ennis L , Wykes T . Impact of patient involvement in mental health research: longitudinal study. Br J Psychiatry. 2013;203(5):381‐386. https://doi.org/10.1192/bjp.bp.112.119818 24029538 10.1192/bjp.bp.112.119818

